# Genomic and Functional Evaluation of Two *Lacticaseibacillus paracasei* and Two *Lactiplantibacillus plantarum* Strains, Isolated from a Rearing Tank of Rotifers (*Brachionus plicatilis*), as Probiotics for Aquaculture

**DOI:** 10.3390/genes15010064

**Published:** 2024-01-01

**Authors:** Diogo Contente, Lara Díaz-Formoso, Javier Feito, Pablo E. Hernández, Estefanía Muñoz-Atienza, Juan Borrero, Patrícia Poeta, Luis M. Cintas

**Affiliations:** 1 Grupo de Seguridad y Calidad de los Alimentos por Bacterias Lácticas, Bacteriocinas y Probióticos (SEGABALBP), Sección Departamental de Nutrición y Ciencia de los Alimentos, Facultad de Veterinaria, Universidad Complutense de Madrid, 28040 Madrid, Spain; diogodas@ucm.es (D.C.); lardia01@ucm.es (L.D.-F.); ehernan@ucm.es (P.E.H.); ematienza@ucm.es (E.M.-A.); jborrero@ucm.es (J.B.); lcintas@ucm.es (L.M.C.); 2 Microbiology and Antibiotic Resistance Team (MicroART), Department of Veterinary Sciences, University of Trás-os-Montes and Alto Douro, 5000-801 Vila Real, Portugal; ppoeta@utad.pt; 3 Associate Laboratory for Animal and Veterinary Science (AL4AnimalS), University of Trás-os-Montes and Alto Douro, 5000-801 Vila Real, Portugal

**Keywords:** aquaculture, larviculture, rotifers (*Brachionus plicatilis*), probiotics, lactic acid bacteria, lactobacilli, WGS, bioinformatics, plantaricins

## Abstract

Aquaculture plays a crucial role in meeting the increasing global demand for food and protein sources. However, its expansion is followed by increasing challenges, such as infectious disease outbreaks and antibiotic misuse. The present study focuses on the genetic and functional analyses of two *Lacticaseibacillus paracasei* (BF3 and RT4) and two *Lactiplantibacillus plantarum* (BF12 and WT12) strains isolated from a rotifer cultivation tank used for turbot larviculture. Whole-genome sequencing (WGS) and bioinformatics analyses confirmed their probiotic potential, the absence of transferable antibiotic resistance genes, and the absence of virulence and pathogenicity factors. Bacteriocin mining identified a gene cluster encoding six plantaricins, suggesting their role in the antimicrobial activity exerted by these strains. In vitro cell-free protein synthesis (IV-CFPS) analyses was used to evaluate the expression of the plantaricin genes. The in vitro-synthesized class IIb (two-peptide bacteriocins) plantaricin E/F (PlnE/F) exerted antimicrobial activity against three indicator microorganisms, including the well-known ichthyopathogen *Lactococcus garvieae*. Furthermore, MALDI-TOF MS on colonies detected the presence of a major peptide that matches the dimeric form of plantaricins E (PlnE) and F (PlnF). This study emphasizes the importance of genome sequencing and bioinformatic analysis for evaluating aquaculture probiotic candidates. Moreover, it provides valuable insights into their genetic features and antimicrobial mechanisms, paving the way for their application as probiotics in larviculture, which is a major bottleneck in aquaculture.

## 1. Introduction

Aquaculture has become a critical pillar within the global food supply chain and represents the fastest-growing sector in food production. Considering the expected surge in the human population, which could reach approximately 9.8 billion individuals by the year 2050, as well as the new global demographic dynamics, the demand for food and protein sources is expected to soar. In this context, aquaculture emerges as a vital alternative to meet this growing demand [1,2]. Nevertheless, climate change and the expansion and intensification of aquaculture pose numerous challenges for this sector, including disease outbreaks, the emergence and resurgence of ichthyopathogens (viral, bacterial, and parasitic), and the prevalence of antimicrobial-(multi)resistant bacteria [2,3,4,5,6].

In the past, the indiscriminate and erroneous use of antibiotics in aquaculture was somewhat underestimated. Yet, due to the nature of aquatic environments, the constant surface interaction between water and fish, and the intense nature of aquaculture, these practices can promote the proliferation of multiple pathogenic bacteria, which can act as reservoirs for the transference of antimicrobial resistance genes. Besides, both residues and sedimentation resulting from the administration of medicated feeds containing uneaten and non-absorbed antimicrobial compounds, as well as their metabolites, pose significant contamination threats to aquatic ecosystems [3,5,7].

Innovative and complementary strategies for disease control in aquaculture, including vaccines, prebiotics, probiotics, postbiotics, symbiotics, bacteriophages, and immunostimulants, have emerged to address these growing challenges. These novel strategies are of special need during the larval rearing phases of aquaculture, as they are often affected by impactful infectious disease outbreaks. In particular, probiotics, which can be defined as live microorganisms that confer health benefits when provided in adequate proportions, are regarded as one of the most promising strategies for aquaculture. Although specific guidelines for evaluating and selecting aquaculture probiotics are currently scarce, several authors and institutions have established principles and general guidelines. These recommendations are based on safety assessments of microorganisms used in food and feed, taxonomical identification, and factors such as resistance to antibiotics or virulence factors. In particular, the European Food Safety Authority (EFSA) recently established as a standard protocol the whole-genome sequencing (WGS) of bacterial probiotics intended for use in the food chain and outlined guidelines for its analysis [8,9,10,11,12]. Lactic acid bacteria (LAB) is the principal group of bacteria proposed as probiotics for aquaculture, and their use has gathered increasing attention over the years. This interest is driven by several factors, such as the overall categorization of the qualified presumption of safety (QPS) by the EFSA, their antimicrobial properties (such as the production of bacteriocins, organic acids, hydrogen peroxide, reuterin, carbon dioxide, and acetaldehyde), their competition for adhesion sites and nutrients, and even their immunomodulatory potential [9,13].

Amongst the several challenges faced by the aquaculture sector, larviculture stands as one of its major bottlenecks, in which frequent infectious disease outbreaks and populational crashes in larvae cause serious economic losses. Interestingly, finfish larviculture mostly relies on the use of live feed such as rotifers as food sources for the early larval stages. During this phase, fish larvae have an underdeveloped microbial community and immune system, as they are frequently exposed to high loads of organic matter and bacterial concentrations [9,13]. To this effect, LAB are often suggested as new alternatives to the conventional chemotherapeutical options in aquaculture, such as the use of antibiotics, as well as new biocontrol mechanisms for the industry [10,12,14,15]. Moreover, there is, to a certain degree, a consensus that beneficial bacteria isolated from autochthonous sources (i.e., isolated from the same species, or rearing environment, as its target species) have greater chances of succeeding as probiotic candidates [14]. In this regard, two *Lc. paracasei* and two *Lp. plantarum* strains, previously isolated by our group from the rearing tank of rotifers (*Brachionus plicatilis*) used as live feed for turbot (*Scophthalmus maximus*, L.) larvae, showed a broad and strong antimicrobial activity spectrum against several bacterial ichthyopathogens of importance to turbot larviculture. Moreover, an in vitro safety assessment indicated their safety, as well as the absence of antibiotic resistance genes and virulence factors [16].

Considering the probiotic potential demonstrated by these four LAB strains, their whole-genome sequences were determined and further analyzed through a bioinformatic (in silico) analysis. Additionally, given the strong antimicrobial properties previously demonstrated by these probiotic candidates and the need to gain further insight into their antimicrobial mechanisms, in vitro cell-free protein synthesis (IV-CFPS) and colony matrix-assisted laser desorption/ionization time-of-flight mass spectrometry (MALDI-TOF MS) analyses were performed.

## 2. Materials and Methods

### 2.1. Growth Conditions and Genomic DNA Isolation

*Lp. plantarum* (BF12 and WT12) and *Lc. paracasei* (BF3 and RT4) strains were grown in de Man, Rogosa, and Sharpe (MRS) agar (1.5%, *w*/*v*) plates (Oxoid, Basingstoke, UK) at 30 °C overnight.

For total genomic DNA isolation, the NZY Microbial gDNA Isolation Kit (NZYTech, Lisbon, Portugal) was used according to the manufacturer instructions. The final eluate was stored at −20 °C until further use.

### 2.2. Whole-Genome Sequencing, Assembly, and Mapping

The whole-genome sequencing (WGS) of the four probiotic candidates was performed at the SeqCenter (Pittsburgh, PA, USA). Briefly, libraries were prepared using the Illumina DNA Prep kit and Integrated DNA Technologies (IDT) 10 bp unique dual index (UDI) indices. Then, they were sequenced on an Illumina NextSeq 2000 (Illumina, San Diego, CA, USA), producing 2 × 150 bp reads. Demultiplexing, quality control, and adapter trimming were performed with a BCL Convert v3.9.3 (Illumina). The resulting sequence reads were assembled into contigs using the Unicycler v0.4.8 program [17]. Rounds of assembly polishing were carried out with the Pilon program (Oxford Nanopore Technologies, Oxford, UK). Additionally, genome maps of the four probiotic candidates were generated using the Proksee web server (https://proksee.ca/, accessed on 13 September 2023). Finally, some genomic features of the four probiotic candidates were compared with those of the species type strains *Lc. paracasei* JCM8130 and *Lp. plantarum* DSM20174.

### 2.3. Bioinformatic (In Silico) Analysis

#### 2.3.1. Species Identification

Although the strains were previously taxonomically identified through DNA sequencing of the PCR-amplified gene encoding the 16S rRNA subunit (*16SrDNA*) as *Lp. plantarum* (BF12 and WT12) and *Lc. paracasei* (BF3 and RT4), their identity was confirmed by two different databases: SpeciesFinder v.2.0. (https://cge.food.dtu.dk/services/Speci esFinder/, accessed on 12 September 2023) and KmerFinder v.3.0.2. (https://cge.food.dtu.dk/services/KmerFinder/, accessed on 13 September 2023). These servers predict bacterial identity based on the complete sequence of the *16SrDNA* and on the number of concurrent kmers (namely, 16-mers), respectively [18,19]. Additionally, a phylogenetic whole-genome sequencing-based mapping was obtained through the Type (Strain) Genome Server (TYGS) (https://tygs.dsmz.de/, accessed on 13 December 2023).

#### 2.3.2. Transferable Antibiotic Resistances

A BLASTn search against the ResFinder tool v.4.3.3. database (http://genepi.food.dtu.dk/resfinder, accessed on 13 December 2023) [20] was performed to identify the acquired antimicrobial resistance genes in the four probiotic candidates.

#### 2.3.3. Virulence Factors and Pathogenicity

A BLASTn search was run against the VirulenceFinder v.2.0.3 database (https://cge.food.dtu.dk/services/VirulenceFinder/, accessed on 13 December 2023). This server finds and predicts genes encoding for bacterial virulence factors [21]. Furthermore, another BLASTn search run to assess the virulence and pathogenicity towards humans using the PathogenFinder v.1.1. web server (https://cge.food.dtu.dk/services/Pathogen Finder/, accessed on 13 December 2023) [22] was carried out.

#### 2.3.4. Mobile Genetic Elements (MGEs)

Likewise, a BLASTn search was performed against the MobileElementFinder database v1.0.2. (https://cge.food.dtu.dk/services/MobileElementFinder/, accessed on 13 December 2023) in search of intracellular MGEs—specifically, insertion sequences (ISs), as well as extracellular MGEs such as plasmids and prophages [23].

#### 2.3.5. Probiotic Traits

A manual prospection of coding sequences (CDSs) was carried out by using the online server Rapid Annotation using Subsystem Technology (RAST) and SEED v.2.0 (http://rast.nmpdr.org/, accessed on 2 October 2023) [24]. The probiotic traits investigated included factors related to adhesion and aggregation, amino acid metabolism, the production of lactic acid, active metabolism, vitamin biosynthesis, stress tolerance, host gastrointestinal tract adaptations, and enzyme production for food digestion.

#### 2.3.6. CRISPR/CRISPR-Cas

The CRISPRCasFinder server v.1.1.2. (https://crisprcas.i2bc.paris-saclay.fr/CrisprCasFinder/Index, accessed on 13 December 2023) [25] was used to predict clustered regularly interspaced short palindromic repeats (CRISPR) and CRISPR-associated genes (*cas*).

#### 2.3.7. Bacteriocin Production

To understand the potential antimicrobial mechanisms of the four probiotic candidates, bacteriocin mining was performed through the BAGEL v4.0. online webserver (http://bagel4.molgenrug.nl/accessed on 13 December 2023) [26].

### 2.4. In Vitro Cell-Free Protein Synthesis (IV-CFPS) of Bacteriocins

Total genomic DNA from *Lp. plantarum* BF12 and WT12 was used for PCR-amplification of the genes encoding the mature bacteriocins of interest. The oligonucleotide primers (Table 1) were obtained from Thermo Fisher Scientific (Waltham, MA, USA) based on primers previously described and used [27]. Phusion Hot Start II High-Fidelity DNA Polymerase (Thermo Fisher Scientific) was used for PCR-amplification in 50 μL reaction mixtures containing 1 μL of genomic DNA. PCR-derived amplicons were visualized via agarose (1.5%, *w*/*v*) gel electrophoresis, dyed with GelRed Nucleic Acid Gel Stain (Biotium, Inc., Fremont, CA, USA) in a ChemiDoc Imaging System (Bio-Rad, Hercules, CA, USA), and quantified using a Qubit fluorometer system (Invitrogen, Waltham, MA, USA). For in vitro cell-free protein synthesis (IV-CFPS) reactions, the PCR-derived amplicons (standardized at a final DNA concentration of 10 ng/µL) were used as templates by using a PURExpress In vitro Protein Synthesis Kit (New England Biolabs, Ipswich, MA, USA) according to the manufacturer protocol [28].

### 2.5. Antimicrobial Activity of In Vitro Synthesized Bacteriocins

The antimicrobial activity of the in vitro-synthesized bacteriocins was evaluated by a spot-on-agar test (SOAT) [29]. Briefly, 10 μL of the samples were spotted onto MRS agar (1.5%, *w*/*v*) plates, which were then overlaid with a soft agar (0.8%, *w*/*v*) previously seeded with ca. 10^5^ CFU/mL of fresh overnight cultures of the indicator microorganisms (*Aeromonas salmonicida* CLFP23, *L. garvieae* CLG4, *Listeria seeligeri* CECT917, *Staphylococcus pseudintermedius* ICM21/02217, *Streptococcus parauberis* LMG225, and *Vibrio anguillarum* CECT4344) (Table S1). After incubation, the growth inhibition zones were analyzed and measured.

### 2.6. Colony Matrix-Assisted Laser Desorption/Ionization Time-of-Flight Mass Spectrometry (MALDI-TOF MS) of Lp. plantarum BF12 and Lp. plantarum WT12

Colony MALDI-TOF MS analysis of both *Lp. plantarum* BF12 and *Lp. plantarum* WT12 was performed as previously described by Lawrence et al. [30], with slight modifications. Briefly, single colonies of the strains grown on MRS agar plates (1.5%, *w*/*v*) at 30 °C for 48 h were picked and resuspended in 50 μL 100% (*v*/*v*) isopropanol with 0.1% (*v*/*v*) trifluoroacetic acid (TFA). The mixtures were vortexed and centrifuged at 11,000× *g* for 30 s. Subsequently, 1 μL of the corresponding supernatant was mixed with 1 μL of a sinapic acid matrix (Sigma-Aldrich, St. Louis, MO, USA) in 30% (*v*/*v*) acetonitrile and 0.3% (*v*/*v*) TFA. Then, the mixtures were transferred onto the MS target plate and dried. The MALDI-TOF MS analysis of samples was conducted on an Ultraflex workstation (Bruker Daltonics, Billerica, MA, USA), equipped with a 337 nm nitrogen laser, at the Unidad de Espectrometría de Masas (CAI Técnicas Químicas, Universidad Complutense de Madrid, Madrid, Spain). The mass spectrometer was calibrated with protein calibration standard I (4000–20,000 *m*/*z*) according to the manufacturer instructions. The FlexControl Software v.2.4. (Bruker Daltonics) was used for sample analysis and control of method parameters.

## 3. Results and Discussion

### 3.1. Whole-Genome Sequencing, Assembly, and Mapping

**Figure 1:**
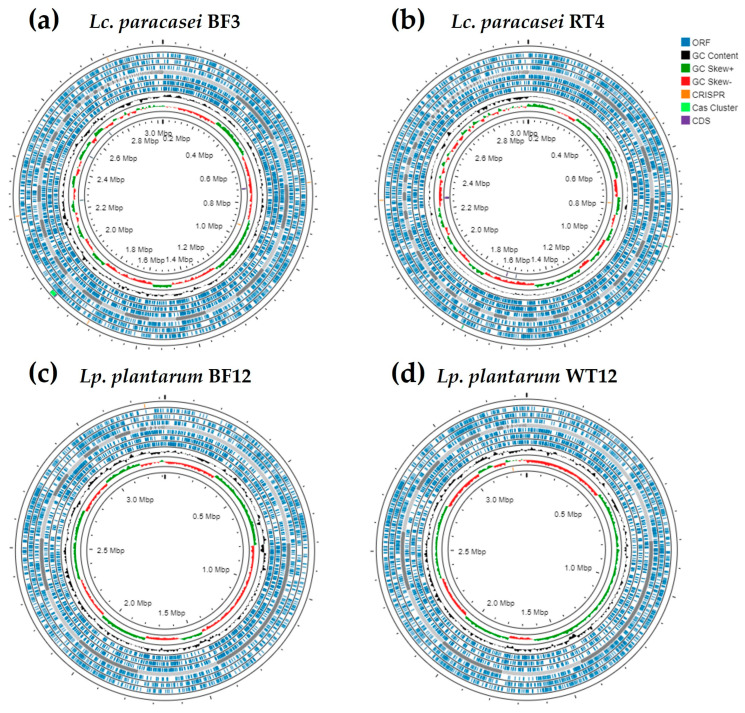
Genome maps of the genomes of (**a**) *Lc. paracasei* BF3, (**b**) *Lc. paracasei* RT4, (**c**) *Lp. plantarum* BF12, and (**d**) *Lp. plantarum* WT12 generated using the Proksee web server [31]. The blue, black, dark green, and red regions represent the open reading frames (ORFs), the GC content, and the CG skew + and −, respectively. Additionally, in the outer layer, orange, light green, and purple zones represent CRISPR sequences, *cas* clusters, and their respective CDSs, respectively.

The size (expressed in bp), number of contigs, G + C content, L50 and N50 values, and number of RNAs of the whole-genome sequence of *Lc. paracasei* (BF3 and RT4) and *Lp. plantarum* (BF12 and WT12), as well as their respective species type strains, are shown in Table 2. Additionally, the genome maps of these four strains, generated using the Proksee web server, are shown in Figure 1.

### 3.2. Bioinformatic (In Silico) Analysis

#### 3.2.1. Species Identification

The SpeciesFinder v.2.0. software confirmed the previous taxonomic identification of the four probiotic candidates as two *Lp. plantarum* (BF12 and WT12) and two *Lc. paracasei* (BF3 and RT4) [17]. Moreover, the KmerFinder v.3.0.2. revealed a 95,565 and 95,567 kmer matching of *Lp. plantarum* BF12 and WT12 with the reference strain *Lp. plantarum* ZDY2013, respectively. Moreover, this software matched (88,322 kmers) *Lc. paracasei* RT4 with the reference strain *Lc. paracasei* WX322. Nevertheless, the KmerFinder v.3.0.2. matched *Lc. paracasei* BF3 with the two reference strains, *Lc. casei* BL23 and *Lc. paracasei* MGB0245 (98,073 and 9110 kmers, respectively). This inconclusive result could be due to the close genetic relatedness between the species *Lc. paracasei* and *Lc. casei*, which can lead to cross-identification, as previously reported [32,33].

**Figure 2:**
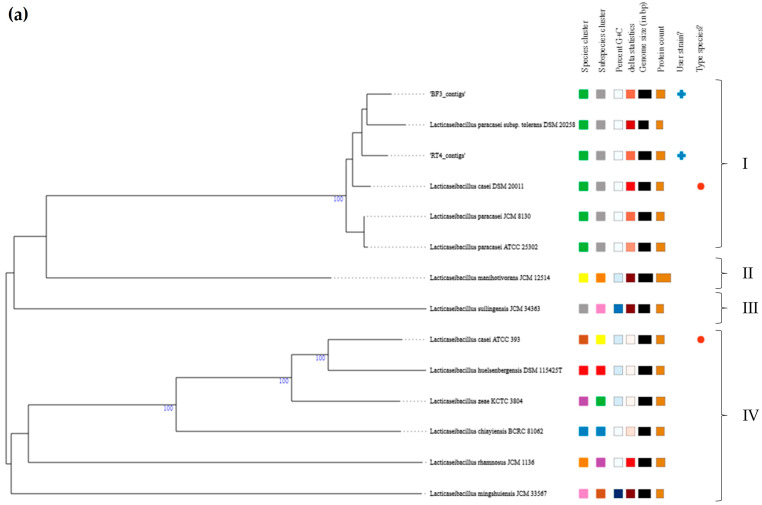

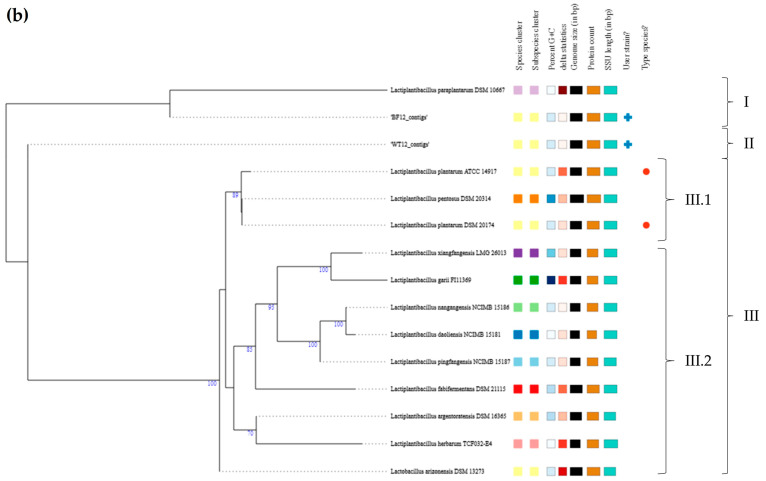
WGS-based phylograms of (**a**) *Lc. paracasei* BF3 and RT4 and (**b**) *Lp. plantarum* BF12 and WT12, generated using the TYGS webserver [35]. Blue crosses and red dots indicate the probiotic candidate strains and the species type strains, respectively.

The TYGS phylogenetic predictions for the probiotic candidate *Lc. paracasei* strains (Figure 2a) revealed four highly divergent phylo-clusters (I to IV). Our strains, *Lc. paracasei* BF3 and RT4, are both closely phylogenetically related to *Lc. paracasei* subsp. tolerans DSM20258, and, together with the type strain *Lc. casei* DSM20011, as well as the other *Lc. paracasei* strains, constituted phylo-cluster I. The inclusion of *Lc. paracasei* and *Lc. casei* strains in the same phylo-cluster is not surprising, since, as stated above, both species are closely genetically related [33,34]. On the other hand, the TYGS phylogenetic predictions for the probiotic candidate *Lp. plantarum* strains showed three divergent phylo-clusters (I to III) (Figure 2b). Interestingly, *Lp. plantarum* BF12 and *Lp. plantarum* WT12 were grouped in two highly divergent phylo-clusters (I and II, respectively), with both being poorly phylogenetically related to phylo-cluster III, which included the two species type strains (sub-phylo-cluster III.1), as well as the remaining Lactiplantibacillus spp. strains (sub-phylo-cluster III.2). The inclusion of *Lp. plantarum* BF12 within the same cluster as *Lp. paraplantarum* DSM10667 is not surprising, as both species are closely genetically related, with both often being included in the broad *Lp. plantarum* group [34].

#### 3.2.2. Transferable Antibiotic Resistances

The assessment of the absence of transmissible antibiotic resistance genes is of utmost importance for selecting probiotic strains, as they are one of the biggest threats to public health, human and veterinary medicine, and the environment. Even though LAB are usually granted QPS status by the EFSA, screening for transferable antimicrobial resistances is fundamental when considering potential bacterial probiotic candidates for the food and feed chains [11,36,37,38]. In this context, the BLASTn searches performed against the ResFinder v.4.3.3. database confirmed the absence of transferable antibiotic resistances in the four probiotic candidates. These results are in accordance with those previously found by determining the minimum inhibitory concentration (MIC) of 12 relevant antibiotics through broth microdilution testing [16]. Furthermore, antibiotic resistance genes were also not detected in the species type strains *Lc. paracasei* JCM8130 or *Lp. plantarum* DSM20174.

#### 3.2.3. Virulence Factors and Pathogenicity

The potential use of LAB strains as probiotics is also dependent on the assessment of other safety traits, such as the absence of virulence factors and pathogenicity, which should be assessed with both in vitro and in silico approaches [11,39,40]. Regarding this, the BLASTn searches against the VirulenceFinder v.2.0.3 database revealed no matches for the four potential probiotic strains, thus confirming the absence of virulence factors, which is in agreement with our previous in vitro safety studies [16]. Likewise, no hits were detected for either species type strain. Additionally, the PathogenFinder v.1.1. software predicted the four probiotic candidates, as well as their respective species type strains, as non-human pathogen microorganisms (Table 3).

#### 3.2.4. Mobile Genetic Elements (MGE)

The MobileElementFinder v1.0.2. software did not identify prophages or plasmids in any of the four probiotic candidates. Nevertheless, it did identify the presence of at least one true IS (e-values of 0.0) [11] for each candidate strain (Table 4).

The absence of both plasmids and prophages can be considered an additional positive trait for probiotic candidates. In this respect, plasmids are regarded as important mechanisms for the transference of antimicrobial resistance genes, which may lead to the generation of multi-drug-resistant bacteria. For instance, plasmid-mediated mechanisms are especially responsible for the spread and production of extended-spectrum β-lactamases (ESBLs) [41]. Similarly, prophages have been associated with some harmful cellular processes, such as antibiotic (multi)resistances, virulence, and even deleterious metabolic pathways [11,42].

On the other hand, all the predicted ISs were transposases, most of which have an undetermined function, and, similar to those found in microorganisms, are generally regarded as potential probiotic LAB. Interestingly, some of the predicted ISs, namely ISLpl1 and ISP1, were shared by the *Lp. plantarum* and *Lc. paracasei* strains, which were isolated from the same rotifer rearing tank. In this context, it is not uncommon to observe the horizontal transference of ISs as part of a co-habitational adaptive evolution [43,44].

#### 3.2.5. Probiotic Traits

The probiotic traits identified in the four probiotic candidates using the RAST web server included genes involved in adhesion and aggregation, the production of lactic acid, survival under stressful conditions and in the host gastro-intestinal tract, and amino acid metabolism and biosynthesis.

The ability to adhere to the host gastrointestinal tract is one of the most desired probiotic traits for bacterial candidates, being a fundamental prerequisite step towards gut colonization [45,46]. Regarding this, several genes hypothesized to encode surface proteins, such as enolase, sortase A, and tyrosine–protein kinases (EspC and EspD), were identified in all of the *Lc. paracasei* and *Lp. plantarum* strains. Furthermore, genes encoding triosephosphate isomerase were found in both *Lc. paracasei* strains, whereas the exopolysaccharide (EPS) biosynthesis gene was predicted for both *Lp. plantarum* strains. Sortase A, for instance, has been described as a relevant surface protein for the adhesion and colonization of several lactobacilli in porcine in vitro models [47]. Moreover, surface proteins expressed by lactobacilli have demonstrated positive immunomodulatory effects on the midgut of white-leg shrimp (*Litopenaeus vannamei*) [48].

LAB attract interest from the food technology sectors mostly due to their lactic fermentation metabolism, meaning that they produce lactic acid as an end-product. LAB can produce both enantiomer forms of lactic acid (D- and L-lactic acid), which are synthesized by the D-lactate and L-lactate dehydrogenases, respectively. Interestingly, the RAST analysis detected the presence of both D- and L-lactate dehydrogenase genes in the four probiotic candidates, a feature frequently reported for lactobacilli and that might be an advantageous probiotic trait [49]. Several recent studies focusing on both human and veterinary medicine have highlighted the antimicrobial importance and role of lactic acid against relevant bacterial pathogens [50,51].

The suitability of probiotic candidates heavily relies on their ability to withstand the stressful conditions of the surrounding environment and the host gastro-intestinal tract, including extreme temperatures, oxidative stress, low pH, and high bile salt concentrations. Regarding this, the RAST software predicted in all of the *Lc. paracasei* and *Lp. plantarum* strains the presence of genes encoding classic heat-shock proteins such as GrpE and DnaK [52], pyruvate kinases involved in acid resistance [53], and choloylglycine hydrolase and CTP synthase, which confer resistance to bile salts [54,55]. Moreover, several osmoprotectant uptake systems that belong to the opu family were detected in the four probiotic candidates. These uptake systems grant the candidate strains tools to tolerate severe osmotic stress and could also be involved in enduring low- and high-temperature conditions [56]. Lastly, several genes involved in oxidative stress tolerance were predicted, namely, glutathione, pyruvate, and thiol peroxidases [11,55].

Rotifers and marine copepod cultures frequently suffer multiple nutrient limitations (also called co-limitations) that have significant impacts on their growth, fecundity, and reproduction rates. Currently, there is a growing interest in the amino acidic imbalances of rotifer diets, which are ultimately used as live feed in marine larviculture. It has been suggested that the dietary amino acid content, particularly of histidine, isoleucine, arginine, and leucine, are of special relevance for rotifer nutrition [57,58]. Regarding this, the RAST software predicted that all of the probiotic candidates possess multiple genetic tools involved in the biosynthesis pathways of several essential amino acids, such as histidine and isoleucine, as well as lysine and methionine. Several authors have reported several beneficial effects of feed supplementation with amino acids or amino acid-producing probiotics in finfish and shrimp aquaculture [59,60]. Regarding this, recent studies have reported, through LAB supplementation, the positive influence of and increase in amino acidic levels in rainbow trout (*Oncorhynchus mykiss*, Walbaum) serum and feces [61], as well as in muscle amino acid composition of Asian seabass (*Lates calcarifer*) [62].

#### 3.2.6. CRISPR/CRISPR-Cas

The CRISPR-Cas systems are complex adaptive immunity mechanisms present in Archaeaand bacteria that prevent the integration of exogenous DNA (mostly MGE). Although they are thought to be more common in archae, some reports revealed that around 40% of the bacteria possess CRISPR-Cas loci. The CRISPRCasFinder software failed to predict CRISPR arrays in both *Lp. plantarum* strains. Likewise, no CRISPR arrays were predicted for the species type strain *Lp. plantarum* DSM20174. Nonetheless, in both *Lc. paracasei* strains, there was a match for an apparently functional CRISPR-Cas system, with an evidence level of 4 (the highest possible) and the presence of two cas clusters: (i) a general class II cluster with cas9, cas1, and csn2 additional proteins and (ii) a general class I cluster with a cas2 additional protein. On the contrary, no CRISPR arrays were detected for the species type strain *Lc. paracasei* JCM8130. Overall, CRISPR-Cas systems have a modular organization that consists of three mandatory processes (adaptation, expression, and interference, which includes the target cleavage) and a signal transduction/ancillary process present only in certain types of the class I systems. The systems predicted for both *Lc. paracasei* strains contained the high-evidence-level CRISPR array, the Cas1 and Cas2 proteins required for the adaptation process, and the Cas9 protein involved in expression and interference events [63]. Altogether, both *Lc. paracasei* strains have the genomic and proteomic tools for the presence of a functional class II type II CRISPR-Cas system. Finally, it should be highlighted that the RAST analysis also revealed several genes encoding CRISPR-associated proteins, strengthening the existence of a functional CRISPR array in *Lc. paracasei* BF3 and RT4.

#### 3.2.7. Bacteriocin Production

**Figure 3:**
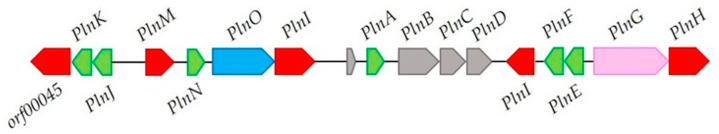
Predicted genetic organization of the gene cluster encoding production of plantaricins A, E/F, J/K, and N in *Lp. plantarum* BF12 and *Lp. plantarum* WT12 using the BAGEL v.4.0. software [26]. Open reading frames (ORFs) are shown by arrows, and those with a known function are indicated by gene identification.

The BAGEL v4.0. software, used for bacteriocin mining, predicted the presence of a multi-bacteriocinogenic gene cluster in the first contig of each *Lp. plantarum* strain (Figure 3). This cluster consisted of operons similar to those first described in *Lp. plantarum C11*, a bactericidal LAB isolated from natural cucumber fermentations [26,27,64,65,66]. According to these previous studies, this cluster encodes the production of several class II bacteriocins, namely, plantaricins (Pln) (PlnA, E/F, J/K, and N), which traditionally should include 21 well-described genes organized in five operons: plnABCD, plnEFI, plnJKLR, plnMNOP, and plnGHSTUV.

Briefly, the operon plnABCD consists of a bacteriocin-like peptide named plantaricin A, encoded by plnA, which acts as a bacteriocin but also as an induction factor. The rest of the operon consists of a histidine protein kinase (HPK) encoded by plnB and two cytoplasmatic response regulators (RR) encoded by plnC and plnD. The operons plnEFI and plnJKLR encode two small cationic bacteriocin-like peptides, namely, plantaricins E/F and J/K, respectively. Both operons are followed by open reading frames (ORFs) encoding cationic hydrophobic peptides similar to bacteriocin immunity proteins (plnI and plnL/R, respectively). The BAGEL v.4.0. software did not predict the existence of any ORF similar to plnL or plnR. However, it predicted the existence of an ORF encoding an immunity protein right before the operon plnJK (orf00045) and predicted plnI. On the other hand, on the operon plnMNOP, the plnN encodes a bacteriocin-like peptide called plantaricin N. PlnN should be followed by two putative immunity proteins, which are encoded either by plnM or by plnP (which was not detected by the bacteriocin-mining software). Nevertheless, previous reports demonstrated that plnP is physiochemically and structurally very similar to plnI, which, in this case, was predicted twice for both strains. In light of this, it is possible to theorize that the plnI closer to the operon plnMNOP could be encoding a bacteriocin immunity protein similar to plnP. Lastly, the operon plnGHSTUV contains two ORFs that encode an ATP-binding casssette (ABC) transporter and its accessory protein (plnG and plnH, present in *Lp. plantarum* BF12 and WT12). These proteins are involved in processing and exporting peptides with double glycine-type leaders, such as PlnA, PlnE, PlnF, PlnJ, PlnK, and PlnN [27,64,65,66]. It should be noted that the functions of plnS, plnT, plnU, and plnV are yet to be fully understood. The production of plantaricins, namely, PlnA, PlnE/F, PlnJ/K, and PlnN, by several candidate probiotic strains of *Lp. plantarum* has previously been described [64,65,66,67,68,69,70]. In this regard, the same multi-bacteriocinogenic gene cluster found in *Lp. plantarum* BF12 and *Lp. plantarum* WT12 was encountered in the species type strain *Lp. plantarum* DSM20174.

On the other hand, the BAGEL v.4.0. software failed to predict the existence of any bacteriocinogenic gene cluster for *Lc. paracasei* BF3, *Lc. paracasei* RT4, or the species type strain *Lc. paracasei* JCM8130.

### 3.3. In Vitro Cell-Free Protein Synthesis (IV-CFPS) and Antimicrobial Activity of Plantaricins A, E/F, J/K, and N

**Figure 4:**
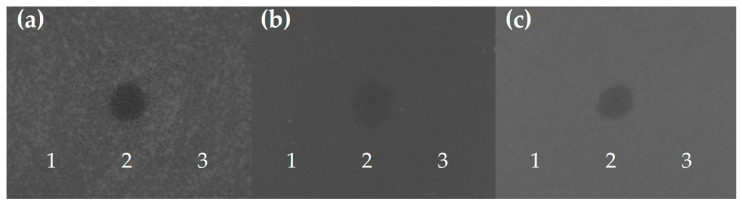
Antimicrobial activity of in vitro-synthesized plantaricins E (1) and F (3) independently and combined in a ratio of 1:1 (2), as determined by using a SOAT against (**a**) *L. garvieae* CLG4, (**b**) *Ls. seeligeri* CECT917, and (**c**) *S. pseudintermedius* ICM21/02217.

To determine the functionality of PlnA, PlnE/F, PlnJ/K, and PlnN, predicted to be encoded by *Lp. plantarum* BF12 and WT12, the antimicrobial activity of the in vitro-synthesized bacteriocins was evaluated by a SOAT (Figure 4). The in vitro-synthesized peptides PlnE and PlnF tested separately did not show antimicrobial activity against any of the microorganisms used as indicators; however, both peptides combined in a ratio of 1:1 exerted antimicrobial activity against the widely known ichthyopathogen *L. garvieae* CLG4, as well as against *Ls. seeligeri* CECT917 and *S. pseudintermedius* ICM21/02217. These results are in accordance with previous reports, since PlnE/F is a class IIb bacteriocin that requires the presence of both peptides in equal molar concentrations to exert antimicrobial activity [67,68,71,72]. However, none of the other in vitro-synthesized plantaricins from *Lp. plantarum* BF12 and WT12 showed antimicrobial activity against any of the tested indicator microorganisms (results not shown).

*Lp. plantarum* and other LAB produce several antimicrobial compounds, mainly bacteriocins and organic acids (e.g., lactic acid, citric acid, acetic acid, and butyric acid), as well as hydrogen peroxide, reuterin, carbon dioxide, and acetaldehyde, which is a desirable probiotic trait [9,13,67,68,72,73,74]. Moreover, multi-bacteriocin production has been widely reported for several lactobacilli and other LAB [29,65,67,68]. In this regard, the prediction of a multi-bacteriocinogenic gene cluster in *Lp. plantarum* BF12 and WT12 led to the hypothesis that the antimicrobial activity exerted by these strains may be due to the production of more than one plantaricin. Nevertheless, as mentioned above, the only in vitro-synthesized plantaricin that demonstrated antimicrobial activity was PlnE/F. Altogether, our results demonstrate that the broad antimicrobial spectrum exerted by *Lp. plantarum* BF12 and WT12 [16] is at least partially due to the production of PlnE/F.

### 3.4. Colony Matrix-Assisted Laser Desorption/Ionization Time-of-Flight Mass Spectrometry (MALDI-TOF MS) of Lp. plantarum BF12 and Lp. plantarum WT12

**Figure 5:**
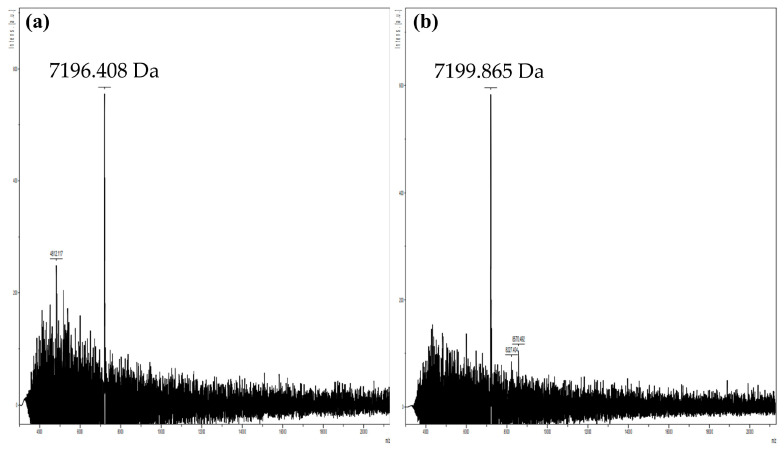
MALDI-TOF MS of (**a**) *Lp. plantarum* BF12 and (**b**) *Lp. plantarum* WT12 cultures.

MALDI-TOF MS analyses of isopropanol-derived extracts from *Lp. plantarum* BF12 and *Lp. plantarum* WT12 cultures grown in MRS agar (1.5%, *w*/*v*) plates revealed the presence of a peptide with a molecular mass of 7196 and 7199 Da, respectively (Figure 5).

Considering the results of the bacteriocin-mining predictions using the BAGEL v4.0. software, as well as the antimicrobial activity of the in vitro-synthesized PlnE/F, the two peaks registered through MALDI-TOF MS analyses could correspond to the dimer form of PlnE/F. The class IIb bacteriocin PlnE/F consists of the 33- and 34-residue peptides PlnE and PlnF, respectively. The theoretical molecular mass of PlnE and PlnF is 3450 and 3700 Da, respectively [71,74,75]. Therefore, the dimer form of PlnE/F should have a theoretical total molecular mass of 7150 Da. The detected peptides had a molecular mass that differed from the theoretical values of PlnEF by 46 Da for *Lp. plantarum* BF12 and by 49 Da for *Lp. plantarum* WT12, which could have been due to the oxidation of three amino acids, which is a phenomenon widely reported for several other bacteriocins [71,74,75,76,77]. Furthermore, our hypotheses are supported by a previous study that showed, through nuclear magnetic resonance spectroscopy, the tridimensional structure of PlnE and PlnF. Moreover, the spontaneous, stable, and preferred dimeric molecular structure of PlnE/F was demonstrated by molecular dynamics simulation [71,78]. On the other hand, none of the other bacteriocins predicted for *Lp. plantarum* BF12 or *Lp. plantarum* WT12 by the BAGEL v.4.0. software were identified by MALDI-TOF MS. Although this technique has been previously reported as a successful method for the detection of class I and II bacteriocins, the absence of the other expected plantaricin peaks in our work could be attributed to the lack of bacteriocin production or the presence of molecular biomarkers produced by these strains, or even culture medium components, which could have masked or suppressed the bacteriocin peptide signals [79,80]. Moreover, some of the bacteriocin genes mined and predicted by the BAGEL v4.0. software and confirmed by IV-CFPS could have been silent genes. Silent genes are DNA sequences frequently found in bacteria that are not normally or fully expressed [81].

## 4. Conclusions

This manuscript emphasizes the importance of the bioinformatic (in silico) analysis of the WGS of probiotic candidates intended for use in the food chain—more specifically, two *Lc. paracasei* and two *Lp. plantarum* strains isolated from a rearing tank of rotifers used as live feed in the larviculture of turbot, which exert a broad and strong antimicrobial activity spectrum against several bacterial ichthyopathogens. Our results confirmed some of the beneficial and safety characteristics previously suggested for these LAB strains through in vitro methods. Moreover, this work allowed for a deeper insight into the genetic features and mechanisms that confer beneficial and probiotic traits on these strains. In this regard, WGS analysis of these strains allowed us to ensure the absence of both transferable antibiotic resistance determinants and genes encoding pathogenic and/or detrimental enzymatic activities and/or virulence factors. Additionally, bacteriocin mining predicted the presence of a multibacteriocinogenic gene cluster encoding six class II bacteriocins (plantaricins) for *Lp. plantarum* BF12 and *Lp. plantarum* WT12, which, to some extent, could be responsible for their antimicrobial activity. However, PlnE/F was the only in vitro-synthesized bacteriocin displaying antimicrobial activity. Furthermore, MALDI-TOF MS analyses of *Lp. plantarum* BF12 and *Lp. plantarum* WT12 cultures detected an extracellular peptide with a molecular mass that matched that of the dimer formed by PlnE and PlnF. In conclusion, the data provided by this study support the use of *Lc. paracasei* BF3, *Lc. paracasei* RT4, *Lp. plantarum* BF12, and *Lp. plantarum* WT12 as probiotic and biocontrol agents in larviculture, one of the major bottlenecks of aquaculture.

## Figures and Tables

**Table 1:** Primers and PCR products used in this study.

Primers and PCR Products	Nucleotide Sequence (5′–3′) ^a^	Amplification Fragment
PlnA-F	**GCGAATTAATACGACTCACTATAGGGCTTAAGTATAAGGAGGAAAAAATATGATG**AAGAGTAGTGCGTATTCTTTGCAGATG	PlnA
PlnA-R	AAACCCCTCCGTTTAGAGAGGGGTTATGCTAGTTACCCATCCCCATTTTTTAAACAGTTTC	PlnA
PlnE-F	**GCGAATTAATACGACTCACTATAGGGCTTAAGTATAAGGAGGAAAAAATATGATG**TTTAATCGGGGCGGTTATAACTTTGGTAAAAG	PlnE
PlnE-R	AAACCCCTCCGTTTAGAGAGGGGTTATGCTAGTTAAAATACCACGAATGCCTGCAACTG	PlnE
PlnF-F	**GCGAATTAATACGACTCACTATAGGGCTTAAGTATAAGGAGGAAAAAATATGATG**GTTTTCCATGCCTATAGCGCGCGTGGC	PlnF
PlnF-R	AAACCCCTCCGTTTAGAGAGGGGTTATGCTAGTTATTCCGTGGATGAATCCTCGGACAG	PlnF
PlnJ-F	**GCGAATTAATACGACTCACTATAGGGCTTAAGTATAAGGAGGAAAAAATATGATG**GGCGCTTGGAAAAATTTCTGGTCTAGTTTAAG	PlnJ
PlnJ-R	AAACCCCTCCGTTTAGAGAGGGGTTATGCTAGTTATACGACGGATTGCTCTGCCAGCTTC	PlnJ
PlnK-F	**GCGAATTAATACGACTCACTATAGGGCTTAAGTATAAGGAGGAAAAAATATGATG**CGTCGGAGTCGTAAAAATGGAATTGGATAC	PlnK
PlnK-R	AAACCCCTCCGTTTAGAGAGGGGTTATGCTAGTTATCTTATTATAATCCCTTGAACCAC	PlnK
PlnN-F	**GCGAATTAATACGACTCACTATAGGGCTTAAGTATAAGGAGGAAAAAATATGATG**AAAAACTATTCTAAGACATGGTGGTAC	PlnN
PlnN-R	AAACCCCTCCGTTTAGAGAGGGGTTATGCTAGTTATACCTAAACCATGCCATGCACTCG	PlnN
**PCR Products**		
PlnA	T7 promoter and transcription terminator containing the mature sequence of the bacteriocin PlnA	
PlnE	T7 promoter and transcription terminator containing the mature sequence of the bacteriocin PlnE	
PlnF	T7 promoter and transcription terminator containing the mature sequence of the bacteriocin PlnF	
PlnJ	T7 promoter and transcription terminator containing the mature sequence of the bacteriocin PlnJ	
PlnK	T7 promoter and transcription terminator containing the mature sequence of the bacteriocin PlnK	
PlnN	T7 promoter and transcription terminator containing the mature sequence of the bacteriocin PlnN	

^a^ T7 promoter is shown in bold; T7 transcription terminator is underlined.

**Table 2:** General characteristics of the WGS of the four bacterial probiotic candidates and their respective species type strains ^a^.

Strains	Size (in bp)	Number of Contigs	G + C Content (in %)	L50 Value	N50 Value	Number of RNAs	tRNA	tmRNA	rRNA	ncRNA	Number of CDS
*Lc. paracasei* BF3	3,031,310	152	46.20	16	59,098	60	46	1	2	11	2907
*Lc. paracasei* RT4	3,031,310	152	46.20	146	59,098	64	49	1	2	12	2883
*Lc. paracasei* JCM8130	2,995,875	1	46.60	-	1	84	61	1	15	7	2924
*Lp. plantarum* BF12	3,319,453	37	44.30	3	343,226	75	65	1	2	7	3138
*Lp. plantarum* WT12	3,340,608	41	44.30	3	343,226	75	65	1	2	7	3161
*Lp. plantarum* DSM20174	3,242,936	1	44.50	-	1	93	72	1	16	4	3016

^a^ Abbreviations: G + C content: content of guanine and cytosine (in %); L50 value: smallest number of sequences whose length makes up 50% of the assembly; N50 value: length of the shortest contig for which longer and equal length contigs cover at least 50% of the total assembly; tRNA: transfer RNA; tmRNA: transfer \–messenger RNA; rRNA: ribosomal RNA; ncRNA: non-coding RNA; CDS: coding sequence.

**Table 3:** Human pathogenicity predictions for the four bacterial probiotic candidates according to the PathogenFinder v.1.1 software [22].

Strain	Probability of Being a Human Pathogen	Matched Pathogenic Families
*Lc. paracasei* BF3	0.101	0
*Lc. paracasei* RT4	0.094	0
*Lp. plantarum* BF12	0.070	0
*Lp. plantarum* WT12	0.070	0

**Table 4:** True insertion sequences (ISs) predicted in the genomes of the four probiotic candidates by the MobileElementFinder v.1.0.2 software [23].

Strains	IS Similar	IS Family	Origin	Length (bp)
*Lc. paracasei* BF3	ISLpl1	IS30	*Lp. plantarum*	1043
	ISPp1	IS30	*Pediococcus pentosaceus*	1039
	ISL1	IS3	*Lc. casei*	1257
	ISLrh3	IS5	*Lc. rhamnosus*	1564
*Lc. paracasei* RT4	ISLrh3	IS5	*Lc. rhamnosus*	1564
	ISLca2	IS5	*Lc. casei*	1563
	ISL1	IS3	*Lc. casei*	1257
*Lp. plantarum* BF12	ISLpl1	IS30	*Lp. plantarum*	1043
	ISPp1	IS30	*P. pentosaceus*	1039
*Lp. plantarum* WT12	ISLpl1	IS30	*Lp. plantarum*	1043
	ISPp1	IS30	*P. pentosaceus*	1039

## Data Availability

The Whole Genome Shotgun projects have been deposited at DDBJ/ENA/GenBank under the accession numbers JAXUFP000000000 (*Lc. paracasei* BF3), JAXUFQ000000000 (*Lc. paracasei* RT4), JAXUFN000000000 (*Lp. plantarum* BF12), and JAXUFO000000000 (*Lp. plantarum* WT12). The versions described in this paper are JAXUFP010000000 (*Lc. paracasei* BF3), JAXUFQ010000000 (*Lc. paracasei* RT4), JAXUFN010000000 (*Lp. plantarum* BF12), and JAXUFO010000000 (*Lp. plantarum* WT12).

## Supplementary Materials

The following supporting information can be downloaded at: https://www.mdpi.com/article/10.3390/genes15010064/s1, Table S1: Growth conditions for the microorganisms used as indicators of the antimicrobial activity of the in vitro-synthesized bacteriocins by a spot-on-agar test.
