# Supplementary material for: Genomic and Functional Evaluation of Two Lacticaseibacillus paracasei and Two Lactiplantibacillus plantarum Strains, Isolated from a Rearing Tank of Rotifers (Brachionus plicatilis), as Probiotics for Aquaculture
Source: Genes (Basel). 2024 Jan 1;15(1):64. doi: 10.3390/genes15010064 (PMC10815930; doi:10.3390/genes15010064)
Supplement: Supplementary file 1 [file genes-15-00064-s001.zip › genes-2730994-supplementary.pdf]

Table S1. Growth conditions for the microorganisms used as indicators of the antimicrobial activity of the in vitro-synthesized bacteriocins by a spot-on-agar test.

Indicator microorganism	Medium[a]	Temperature	Incubation time
Aeromonas salmonicida CLFP23	TSB	25 °C	Overnight
Lactococcus garvieae CLG4	MRS[b]	30 °C	Overnight
Listeria seeligeri CECT917	BHI	37 °C	Overnight
Staphylococcus pseudintermedius ICM21/02217	BHI	37 °C	Overnight
Streptococcus parauberis LMG225	BHI	37 °C	Overnight
Vibrio anguillarum CECT4344	TSB supplemented with NaCl (1.5%, *w*/*v*)	25 °C	Overnight

[a]TSB: Tryptone Soy Broth (Oxoid, Basingstoke, UK); MRS: de Man, Rogosa and Sharpe (Oxoid); BHI: Brain Heart Infusion (Oxoid).
